# Retraction: Screening of drug candidates against Endothelin-1 to treat hypertension using computational based approaches: Molecular docking and dynamics simulation

**DOI:** 10.1371/journal.pone.0284670

**Published:** 2023-04-26

**Authors:** The PLOS ONE Editors

The *PLOS ONE* Editors retract this article [1] because of concerns about peer review and the unlicensed use of Molecular Operating Environment (MOE) software. We regret that the issues were not addressed prior to the article’s publication.

A corresponding author (MQ) acknowledged and apologized for the unlicensed use of this software, and requested that the article be retracted.

MQ agreed with the retraction. MSM and SA disagreed with the retraction. IF, HI, SK, AR, and UAA either did not respond directly or could not be reached.
